# Hydrostatic High-Pressure Post-Processing of Specimens Fabricated by DLP, SLA, and FDM: An Alternative for the Sterilization of Polymer-Based Biomedical Devices

**DOI:** 10.3390/ma11122540

**Published:** 2018-12-13

**Authors:** José A. Robles Linares-Alvelais, J. Obedt Figueroa-Cavazos, C. Chuck-Hernandez, Hector R. Siller, Ciro A. Rodríguez, J. Israel Martínez-López

**Affiliations:** 1 Department of Mechanical Engineering and Advanced Materials, Tecnologico de Monterrey, Monterrey 64849, NL, Mexico; a01226825@tec.mx (J.A.R.L.-A.); obedt.figueroa@tec.mx (J.O.F.-C.); ciro.rodriguez@tec.mx (C.A.R.); 2 Laboratorio Nacional de Manufactura Aditiva y Digital (MADiT), Apodaca 66629, NL, Mexico; 3 Centro de Biotecnología FEMSA, Tecnologico de Monterrey, Monterrey 64849, NL, Mexico; cristina.chuck@tec.mx; 4 Department of Engineering Technology, University of North Texas, 3940 N. Elm. St., Denton, TX 76207, USA; hector.siller@unt.edu

**Keywords:** hydrostatic high-pressure, sterilization, additive manufacturing, FDM, DLP, SLA, biocompatibility, HHP, biomedical devices

## Abstract

In this work, we assess the effects of sterilization in materials manufactured using additive manufacturing by employing a sterilization technique used in the food industry. To estimate the feasibility of the hydrostatic high-pressure (HHP) sterilization of biomedical devices, we have evaluated the mechanical properties of specimens produced by commercial 3D printers. Evaluations of the potential advantages and drawbacks of Fused Deposition Modeling (FDM), Digital Light Processing (DLP) technology, and Stereolithography (SLA) were considered for this study due to their widespread availability. Changes in mechanical properties due to the proposed sterilization technique were compared to values derived from the standardized autoclaving methodology. Enhancement of the mechanical properties of samples treated with Hydrostatic high-pressure processing enhanced mechanical properties, with a 30.30% increase in the tensile modulus and a 26.36% increase in the ultimate tensile strength. While traditional autoclaving was shown to systematically reduce the mechanical properties of the materials employed and damages and deformation on the surfaces were observed, HHP offered an alternative for sterilization without employing heat. These results suggest that while forgoing high-temperature for sanitization, HHP processing can be employed to take advantage of the flexibility of additive manufacturing technologies for manufacturing implants, instruments, and other devices.

## 1. Introduction

Additive manufacturing (AM) has gained popularity in recent years in several medical fields due to the potential to construct prototypes and biomedical devices [1]. Employing AM with biomaterials is promising for the development of customized patient-oriented applications, such as implant fabrication [2] and the development of human organs [3]. While there is a current trend in replacing metals, alloys, and ceramics for polymeric materials, there are special considerations that medical devices should fulfill to perform their function of minimizing the adverse effects of a toxic or immunological response due to the interaction of tissue with the device. Polymers are extensively employed due to their superior attributes, such as their supporting robustness, extended use capacity, and facility to meet specific requirements, such as adhesion or drug release [4].

In general, the sterilization of devices or elements of biomedical prototypes produced with AM has been neglected. However, implants and other long-term or life-supporting apparatus require treatment before coming into contact with human tissue. Compatibility among the manufacturing methodology, the material, and the sterilization method should be assured to comply with both the biocompatibility requirements and the highly-regulated environment. Moreover, regarding the disinfection and sterilization of medical equipment (patient-care items or equipment), a classification of the type of sanitization has been divided into three categories-namely, critical, semi critical, and noncritical-on the basis of the degree of risk of infection involved in the use of the items [5]. The taxonomy comprises items of a broad spectrum of conditions; critical items are those associated with infection if the item is contaminated with any microorganism or spore; semi critical items are those in contact with mucous membranes or nonintact skin, such as endoscopes, anesthesia equipment, or esophageal manometry probes; and noncritical items are those that come in contact with skin, but not mucous membranes (i.e., blood-pressure cuffs, crutches, bed rails, or linens) [6]. The use of this taxonomy has been adopted and promoted by the Centers for Disease Control and Prevention [7]. Due the dissimilar and ubiquitous reach of additive manufacturing, research devices could be considered in any of the aforementioned categories.

Most studies towards the implementation of implants based on additive manufacturing have focused on the effects of the parameters of design, materials, or process. However, personalized additive manufacturing has to deal with the mechanical properties and biocompatibility of the printed material after the sterilization method [8]. Nonetheless, current state-of-the-art and regulations are centered on non-polymeric orthopedic-based materials. The Food and Drug Administration of the United States (FDA) has established dry heat, ethylene oxide, steam, and radiation for orthopedic implants (Class III implants) [9,10]. Although there are other sterilization techniques (i.e., hydrogen peroxide and ozone) that are currently under review, these methods have not been approved by the agency. Additionally, novel sterilization methods, such as pulse light, microwave radiation, and vaporized peracetic acid, have also been reported [11].

Additive manufacturing technologies have shown significant potential for the production of biomedical devices [12]. For example, Point-of-Care (PoC) solutions have shown the potential to be implemented as ultra-low-cost solutions [13,14]. Regardless of the cost-effectivity ratio, the capacity to produce complex structures with polymers is challenged by the mechanical properties of the products and by the limitations that arise from the availability of materials to be processed [15].

The use of HHP as a sterilization method for 3D printed parts has not been reported for biomedical engineering, even though it has been widely used for food preservation [16]. The requirements of an increasing market of health-conscious consumers for high-quality edible products that maintain freshness, nutritional value, and flavor, while decreasing the number of additives employed, have boosted the development of non-thermal preservation technologies, such as high pressure, a pulsed electric field, pulsed light, an electron beam, plasma, and modified atmosphere packaging. Hydrostatic high-pressure (HHP) sterilization does not rely on heat, chemicals, reduced water activity, or reduced temperatures to control pathogens or microbes [17]. Cellular processes are impaired under high-pressure; motility, cell division, and protein synthesis are disabled under 10, 20, and 50 MPa, respectively [18]. High-pressure unfolds proteins, causing the protection provided by the wall membranes to be disrupted until viability is compromised [19]. This technique consists of the appliance of intense pressure (100–1000 MPa) to cause microbial inactivation [16]. HHP has been used extensively to process fruits, vegetables, meat and dairy products.

An HHP system is composed of a vessel and its closure pressure-generation system, temperature control device, and material-handling system. Pressure-transmitting fluids are used in the vessel to transmit pressure uniformly and instantaneously. The samples should be packaged in flexible packaging and loaded in the vessel. A pump or piston is activated to apply the pressure energy and maintained for the desired time; then, the vessel is decompressed. Depending on the application, the compression, holding, de-compression, loading, and unloading can be repeated as required for the application [20]. A recent review of the state of the HHP industry estimated that every year, 500 thousands of tons of products are processed by around 300 industrial machines in production [21].

**Figure 1:**
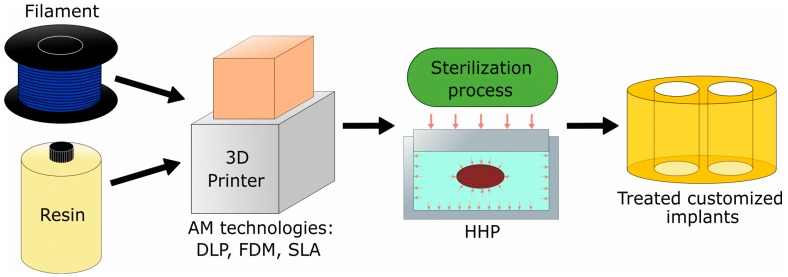
Schematic process for medical devices fabrication (e.g., implants) using AM technologies.

For this paper, we propose high-hydrostatic pressure as a sterilization method to increase the biocompatibility of biomedical devices materials. The design and manufacturing of patient-specific devices is a task of growing interest among the medical community. For example, intervertebral implants have received a lot of attention in consideration of the potential benefits to patients derived from the customization of an implant. Figure 1 illustrates the application of the proposed technology for fabricating an intervertebral implant that could be applied for other medical devices manufactured using additive manufacturing technologies. 

In a recent review, Tipniss and Burgness acknowledged the high sensibility of polymers to the sterilization techniques for polymers, either as a protective coating or while being the substrate of medical devices [11]. Sterilization is defined as a process in which living cells, viable spores, viri, and viroids are eradicated or removed from an object. *Autoclave* (or steam sterilization method) has been considered as a nontoxic, easy to control and monitor, rapid sterilization technology that is available in virtually any medical facility; however, the methodology can be deleterious to heat-sensitive instruments and instruments can be damaged by exposure or rust originating from humidity [6].

The proliferation of additive manufacturing (AM) equipment with a higher resolution has shifted the aim to develop visual prototyping models to end-user parts. Personalized medicine based on tomographic images and the capability to produce pieces with a high complexity have driven the development of a growing interest in additive manufacturing for producing orthopedic implants. However, limitations on the choice of materials, such as the porosity of the parts due to the underlying manufacturing process, mean that there are numerous prototypes of devices with inferior mechanical properties than those fabricated using subtractive or formative technologies [22]. 

There is an inherent demand for high mechanical properties and biocompatibility of AM implants and other medical devices. Espalin et al. [23] proposed the use of Fused Deposition Modeling (FDM) with a medical-grade PMMA filament and found that porosity and fabrication conditions influence the mechanical properties. Domanski et al. [24] proved that Selective Laser Melting (SLM) can produce parts with higher mechanical properties compared to Fused Deposition Modeling (FDM) and Powder Based 3D Printing (3DP). 

Additional to the natural effect of AM as a process, recent research suggests that sterilization procedures may affect the structure, aggregates, and properties of AM materials [25].

Flege et al. [26] studied the effects of different target doses and irradiation of γ-sterilization on the molecular weight of SLM-manufactured materials, but the mechanical properties were not addressed in their evaluation. Béduer et al. [27] proposed an AM technology that is viable for 3D printing scaffolds used in tissue engineering and considered an autoclave sterilization method after printing, but the effects of the sterilization were not quantified. Obaton et al. [28] also considered autoclave sterilization after printing an SLM-processed part, but the effects were not measured.

Following a different approach, Neches et al. [29] proved that it is possible to obtain 3D printed products that are sterilized during the curing process of the material, meaning that no further sterilization procedure is necessary. Furthermore, Zuniga [30] reported the use of FDM for 3D printing sterilized prostheses by using an antibacterial polymer filament.

The study of the effects on the mechanical properties of AM materials due to sterilization techniques could provide insights for improved biocompatibility. This work presents the effects of the mechanical properties of high-hydrostatic pressure (HHP) processing as a sterilization method on samples manufactured with different AM technologies. Three different AM methods are subject to assessment and compared with a standardized autoclave methodology. To evaluate the feasibility of the proposed methodology, we have employed commercially available equipment and reagents. While it would be interesting to examine the suitability of the novel methodology to process different types of risk and devices, and hence the potential to comply with medical policies, we have purposely excluded this discussion.

## 2. Materials and Methods

### 2.1. Additive Manufacturing Technologies and Materials

Three different commercial materials labeled as biocompatible polymeric materials were used for manufacturing the specimens employed to characterize the sterilization process.

A thermoset photopolymer resin (E-Dent 400, Envisiontec, Dearborn, MI, USA) was used in Digital Light Processing (DLP) apparatus (P3 Mini Multilens, Envisiontec). The equipment was loaded with the resin characterized by a yield stress of 80.9 MPa and a Young’s modulus of 2123 MPa. An Otoflash pulse curing chamber from the same supplier was utilized (11 W lamp with a wavelength between 300 and 700 nanometers and 10 pulses per second).

A polycarbonate thermoplastic filament (PC-ISO, Stratasys, Eden Prairie, MN, USA) was processed with Fused Deposition Modeling (FDM) in a Fortus 400mc 3D system by Stratasys. The 3D printer was configured with the maximum resolution of 0.127 mm per layer. 

Another photopolymer (Clear v02, Formlabs, Somerville, MA, USA) was employed in Stereolithography (SLA) 3D printing equipment (Form 1+, Formlabs) employing a 25 μm resolution setting. Samples were cured using the recommended settings for post-processing the sample (15 min at 60 °C and 1 h of exposure to UV light).

**Figure 2:**
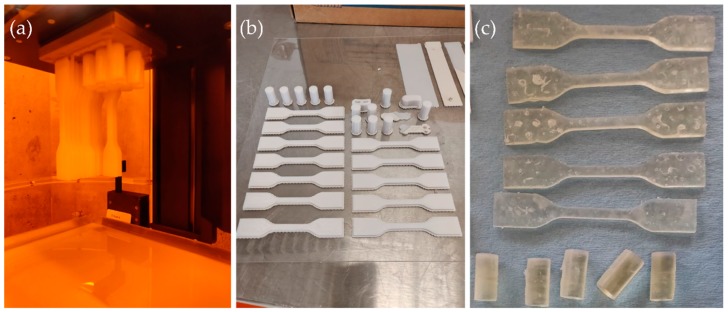
Example of specimens manufactured with (**a**) DLP, (**b**) FDM, and (**c**) SLA technologies.

Each tensile and compressive test sample was manufactured using a common STL file and considering the same printing direction under standard environmental conditions. Figure 2a–c shows examples of some specimens used for evaluation of the DLP, FDM, and SLA technologies, respectively.

### 2.2. Sterilization Methods

Two sterilization methods were performed on two different sets of specimens and compared to a third set of specimens that were not treated.

Hydrostatic high-pressure sterilization was performed in 2 L capacity equipment (HPP, Avure, Middletown, OH, USA) using water as a pressure-transmitting medium. One pressure level (600 MPa) and processing time (15 min) were applied to the samples, which were previously packed in a polyethylene bag and vacuum-sealed.

*Autoclave* sterilization was performed in a 47 L-capacity autoclave (SM510, Yamato Scientific, Santa Clara, CA, USA) with a 15 min cycle program under 121 °C and 0.103 MPa (15 psi). The settings were selected considering standard practices for medical facilities [6,7]. 

### 2.3. Mechanical Testing

To test the mechanical properties of the materials and compare them for different sterilization methods, compressive and tensile tests were conducted.

The tensile tests were performed based on a standardized method [31] considering a Type II specimen. A universal testing machine (3365, INSTRON, Norwood, MA, USA) equipped with a 5 kN load cell was used considering a crosshead speed of 5 mm/min.

The compressive tests were developed following another standardized method [32]. A universal testing machine (AG-250 kN, Shimadzu Scientific Instruments, Columbia, MD, USA) equipped with a 25 kN load cell was used with a crosshead speed of 1.3 mm/min. Cylindrical specimens with a diameter of 12.7 mm and length of 25.4 were used.

The tensile elastic modulus (*E_T_*), compressive elastic modulus (*E_C_*), ultimate tensile strength (*σ_UTS_*), and yield compressive strength (*σ_YCS_*) properties were measured during the tests. In total, 101 samples were used for the experiments.

The yield compressive stress was considered from the data as the offset yield point, which was defined at the strain value of 0.2%. The results were analyzed statistically using one-way ANOVA and the Boferroni Test (α = 0.05) using Origin Pro 2018b (OriginLab, Northampton, MA, USA).

### 2.4. Geometrical and Surface Characterization

**Figure 3:**
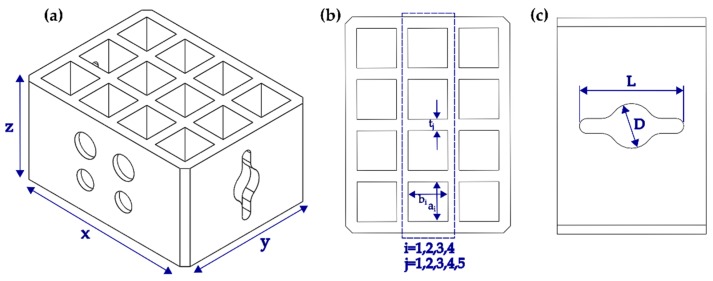
Probes for geometrical characterization: (**a**) overall dimensions; (**b**) top surface; and (**c**) lateral surface details.

To measure plausible deviations in the shape due the proposed post-processing of the samples due to porosity, elastic moduli, and tensile strength in the assessed materials, an additional test probe was designed and manufactured using similar conditions to those described in Section 2.1. A set of one sample and two replicas were considered for a total of nine printed specimens. The testing probe was designed to evaluate changes in the overall height, length, and depth (*x* = 19 mm, *y* = 14.5 mm, *z* = 10 mm; see Figure 3a) for each of the materials before and after the high-hydrostatic pressure treatment. Additional characterization was conducted for changes in the width of sections on the top (a = b = 3.5 mm and t = 1 mm; see Figure 3b) and lateral side of the specimens (*L* = 7 mm, *D* = 3 mm; see Figure 3c). A Mitutyo caliper (500-196-30, Aurora, IL, USA) was employed before and after high hydrostatic pressure treatment with the same conditions described in Section 2.2 for a total of 216 measurements. 

Additional characterization was conducted for a probe manufactured with DLP employing an Alicona surface metrology instrument (InfiniteFocus, Itasca, IL, USA) before and after the hydrostatic high-pressure treatment. The device allows the acquisition of datasets at a high depth of focus, similar to a Scanning Electronic Microscope. IFM has been shown to be capable of capturing images with a lateral resolution down to 400 nm, providing three-dimensional datasets [33]. Test probes produced with FDM and SLA could not be examined properly due the optical properties of the materials. 

### 2.5. Microbiological Screening of the Process

Screening of the presence of Aerobic Mesophilic Bacteria, yeast, and fungi prior sterilization was done by a microbiological culture following the standardized methodology for food analysis [34,35] of a set of specimens. 

## 3. Results

**Figure 4:**
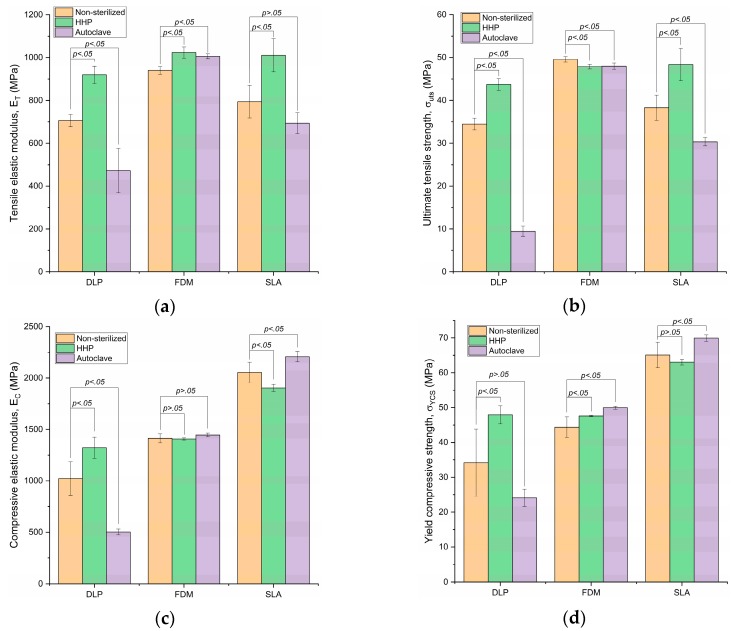
Mechanical properties obtained from the tests for DLP, FDM, and SLA. Mean values, standard deviations, and *p*-values are shown for: (**a**) Tensile elastic modulus (*E_T_*); (**b**) ultimate tensile strength (*σ_uts_*); (**c**) compressive elastic modulus and (*E_C_*); and (**d**) yield compressive strength (*σ_YCS_*).

The results of the tensile and compressive tests are shown in Figure 4 and Table 1. Data are expressed as mean ± SD. Differences were considered as statistically significant when *p* < 0.05.

### 3.1. Tensile Elastic Modulus

Non-sterilized E-Dent 400 (DLP) exhibited an average of 705.8 MPa, with a standard deviation (SD) of 28.4 MPa. The HHP-sterilized *E-Dent 400* mean *E_T_* incremented to 919.6 MPa, with an SD of 40.80 MPa. The autoclave sterilization caused a decrement in the tensile elastic modulus, with an average value of 472.66 MPa and SD of 103.94 MPa.

Regarding the PC-ISO material (FDM), its mean tensile modulus was 940.60 MPa, with an SD of 17.76 MPa, in the non-sterilized variation. The HHP sterilization increased the mean value to 1023.60 MPa, with an SD of 25.88 MPa. The *E_T_* of the autoclave-sterilized PC-ISO was 1006.02 MPa, with an SD of 11.18 MPa.

SLA-processed samples without sterilization showed a mean tensile modulus of 794.31 MPa and SD of 76.17 MPa, while the high-pressure sterilization method exhibited 1011.05 MPa with an SD of 77.23 MPa on this material. The autoclave-sterilized sample had an *E_T_* of 693.86 MPa, with a 48.26 MPa SD.

### 3.2. Ultimate Tensile Strength

Non-sterilized E-Dent 400 (DLP) exhibited a value of 34.48 MPa, with an SD of 1.38 MPa. HHP increased the mean *σ_UTS_* to 43.71 MPa, with an SD of 1.38 MPa. The autoclave sterilization caused a decrement of tensile strength, with an average value of 9.48 MPa and SD of 1.19 MPa.

The FDM-processed PC-ISO mean value of ultimate tensile strength was 49.58 MPa, with an SD of 0.64 MPa, in the non-sterilized variation. The HHP sterilization slightly decreased the mean value to 47.88 MPa, with an SD of 0.56 MPa. The *σ_UTS_* of the autoclave-sterilized PC-ISO was 48.00 MPa, with an SD of 0.73 MPa.

SLA-processed samples without sterilization showed a mean tensile strength of 38.28 MPa and SD of 2.96 MPa, while the HHP sterilization method exhibited a value of 48.37 MPa, with an SD of 3.77 MPa on this material. The autoclave-sterilized variation had a tensile strength of 30.35 MPa, with a 0.96 MPa SD.

### 3.3. Compressive Elastic Modulus

Non-sterilized E-Dent 400 (DLP) exhibited an average of 1022.15 MPa, with an SD of 162.69 MPa. High-pressure-sterilized *E-Dent 400* mean *E_C_* increased to 1322.25 MPa, with an SD of 101.61 MPa. The autoclave sterilization caused a decrement in the compressive elastic modulus, with an average value of 502.53 MPa and SD of 28.66 MPa. Regarding the PC-ISO material (FDM), its mean compressive modulus was 1413.98 MPa, with an SD of 43.09 MPa, in the non-sterilized variation. The HHP sterilization slightly decreased the mean value to 1407.11 MPa, with an SD of 10.08 MPa. The *E_C_* of the autoclave-sterilized PC-ISO was higher, with 1446.27 MPa and an SD of 18.95 MPa. SLA-processed samples without sterilization showed a mean compressive modulus of 2054.57 MPa and SD of 99.18 MPa, while the HHP sterilization method exhibited 1903.29 MPa with an SD of 35.37 MPa on this material. The autoclave-sterilized variation had an *E_C_* of 2207.35 MPa, with a 50.40 MPa SD.

### 3.4. Yield Compressive Strength

Non-sterilized E-Dent 400 (DLP) exhibited an average of 34.19 MPa, with a standard deviation of 9.63 MPa. HHP-sterilized *E-Dent 400* mean *σ_YCS_* increased to 47.91 MPa, with an SD of 2.56 MPa. The autoclave sterilization caused a decrement of compressive yield strength, with an average value of 24.10 MPa and SD of 2.46 MPa. Regarding the PC-ISO material (FDM), its mean value of compressive yield strength was 44.39 MPa, with an SD of 2.98 MPa, in the non-sterilized variation. The HHP sterilization slightly increased the mean value to 47.60 MPa, with an SD of 0.20 MPa. The *σ_YCS_* of the autoclave-sterilized PC-ISO was even higher, with 49.95 MPa and 0.42 MPa SD.

SLA-processed samples without sterilization showed a mean compressive yield strength of 65.09 MPa and SD of 3.68 MPa, while the HHP sterilization method exhibited 63.03 MPa, with an SD of 0.80 MPa on this material. The autoclave-sterilized variation had a strength of 69.95 MPa, with a 0.93 MPa SD.

### 3.5. Geometrical and Surface Characterizations

**Figure 5:**
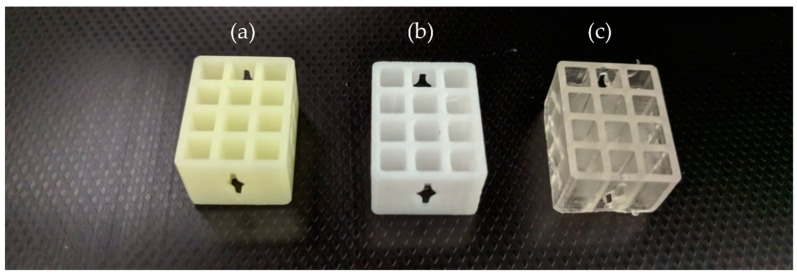
Photos of examples of manufactured and treated probes for geometrical and surface characterization for (**a**) DLP, (**b**) FDM, and (**c**) SLA.

The developed geometrical probes were manufactured using additive manufacturing and treated with the proposed methodology (see Figure 5). The overall variation of the dimensions was −0.02%, 0.04%, and 0.1% considering the data of the DLP, FDM, and SLA probes, respectively. Moreover, the absolute maximum variation of the dimensions after HHP processing was 2.04%, 1.03%, and 2.04% for DLP, FDM, and SLA test probes, respectively. No visible damage was found after HHP processing.

Surface characterization of the Alicona equipment did not show any significant differences. Data of the dimensions is included as Supplementary Materials S1. 

## 4. Discussion

Tensile tests suggest that the HHP sterilization method improves the elastic modulus and the tensile strength (except for the ultimate tensile strength of the FDM-processed material, in which case, it is reduced by 3.43% and shows a *p* > 0.05). According to the data, the sterilization method can increase the elastic modulus by as much as 30.30% on average and the tensile strength by 26.36%.

Unlike HHP, autoclave sterilization had an adverse effect on both tensile properties (except for the tensile elastic modulus of the FDM-processed material, which increased by 6.96%). The tensile elastic modulus decreased by a maximum of 33.03% and the ultimate tensile strength by 72.51%.

In the compressive tests, the DLP-processed material exhibited an increment of 29.40% and 40.12% in the elastic modulus and the compressive yield strength, respectively. Sterilization by the autoclave, in contrast, displayed a noticeable drop in the mechanical properties *E_C_* and *σ_YCS_*. The FDM material with both sterilization methods remained within the standard deviation range of the non-sterilized variation for the compressive elastic modulus. This shows that FDM-processed PC-ISO can withstand autoclave and high-pressure sterilization without any changes to its rigidity. Regarding the yield strength of this material, the autoclave created a significant increment of 12.5% with respect to the non-sterilized material and 4.94% with the high-pressure-sterilized.

In contrast to the tension tests, the SLA material exhibited the greatest compressive elastic modulus and yield compressive strength. The high-pressure process decreased by 7.36% and 3.16% (*E_C_* and *σ_YCS_*, respectively) in comparison with non-sterilized samples. The autoclave process had the opposite effect and increased the values of these properties by 7.44% and 7.47%, respectively. Furthermore, we detected with bare eyes cracks and superficial damages for DLP-processed samples undergoing autoclave sterilization. These irregularities can explain the drastic decrease of the mechanical properties.

Geometrical and surface characterization suggests that the application of high-pressure does not significantly affect the shape of the specimens. This behavior can be explained because the samples are uniformly affected by the pressure transmitting fluid.

Microbiological screening of the specimens before HHP processing suggests that manufacturing processes were developed under Good Laboratory Practices; however, a more thorough evaluation of the presence should be done to evaluate the feasibility of the proposed methodology as a policy complying sterilization alternative. Nonetheless, the augment in the values of the mechanical tensile and compressive properties encourages us to continue researching HHP. The increase in the aforementioned properties could develop better conditions to develop additive manufacturing load bearing medical devices, such as orthopedic implants. Moreover, other devices with manufacturing methods that are constrained to metals or alloys due to the compatibility with a heat-based sterilization technology, could be developed with DLP, FDM, or SLA additive manufacturing technologies.

## 5. Conclusions

The concluding remarks of this work are summarized as follows:

- Hydrostatic high-pressure processing, a method widely used for food preservation, shows the potential to be employed for the sterilization of biomedical devices. It is necessary to evaluate the sanitization capacity of the proposed technology.
- The potential of the technology for cleansing the surface of biomedical devices without employing heat could help overcome the limitation for processing polymer-based medical devices without suffering losses in the mechanical properties, typically associated with heat-based treatments. Additionally, this work has shown that employing a high-pressure sterilization procedure for materials processed under an additive manufacturing process could increase the mechanical properties of some materials. Further research should be conducted to investigate the effects that this sterilization technique has on the microstructure of the polymers.
- Furthermore, it has been proven that PC-ISO processed by FDM technology can withstand both sterilization methods and has a stable mechanical behavior.

## Figures and Tables

**Table 1:** Mechanical properties obtained from the mechanical tests of specimens manufactured with DLP, FDM, and SLA.

Property	AM	Non-Sterilized	HHP		Autoclave	
		Mean ± SD (MPa)	Mean ± SD (MPa) ^1^	Change ^1^	Mean ± SD (MPa)	Change ^2^
$E_{T}$	DLP	705.8 ± 28.37	919.58 ± 40.79 *p* = 3.2 × 10^−4^	30.29%	472.65 ± 103.94 *p* = 1.4 × 10^−4^	−33.03%
	FDM	940.6 ± 17.76	1023.6 ± 25.88 *p* = 1.1 × 10^−5^	8.82%	1006.02 ± 11.18 *p* = 8.7 × 10^−5^	6.95%
	SLA	794.31 ± 76.17	1011.05 ± 77.23 *p* = 1.1 × 10^−3^	27.29%	693.86 ± 48.26 *p* = 14.93 × 10^−2^	−12.65%
$\sigma_{UTS}$	DLP	34.48 ± 1.38	43.71 ± 1.38 *p* = 1.0 × 10^−7^	26.79%	9.48 ± 1.19 *p* = 4.1 × 10^−13^	−72.49%
	FDM	49.58 ± 0.64	47.88 ± 0.56 *p* = 2.3 × 10^−3^	−3.41%	48 ± 0.73 *p* = 2.8 × 10^−3^	−3.18%
	SLA	38.28 ± 2.96	48.37 ± 3.77 *p* = 7.0 × 10^−4^	26.35%	30.35 ± 0.96 *p* = 4.4 × 10^−3^	−20.72%
$E_{C}$	DLP	1022.15 ± 162.69	1322.25 ± 101.61 *p* = 2.9 × 10^−3^	29.36%	502.53 ± 28.66 *p* = 1.7 × 10^−5^	−50.84%
	FDM	1413.98 ± 43.09	1407.11 ± 10.08 *p* = 1.0 × 10^0^	−0.49%	1446.27 ± 18.95 *p* = 27.4 × 10^−2^	2.28%
	SLA	2054.57 ± 99.18	1903.29 ± 35.37 *p* = 1.1 × 10^−2^	−7.36%	2207.35 ± 50.4 *p* = 1.0 × 10^−3^	7.44%
*σ_YCS_*	DLP	34.19 ± 9.63	47.91 ± 2.56 *p* = 9.6 × 10^−3^	40.14%	24.1 ± 2.46 *p* = 6.0 × 10^−2^	−29.51%
	FDM	44.39 ± 2.98	47.6 ± 0.2 *p* = 4.1 × 10^−2^	7.23%	49.95 ± 0.42 *p* = 8.2 × 10^−4^	12.54%
	SLA	65.09 ± 3.68	63.03 ± 0.8 *p* = 53.4 × 10^−2^	−3.15%	69.95 ± 0.93 *p* = 1.5 × 10^−2^	7.47%

^1,2^ In reference to non-sterilized probes.

## Supplementary Materials

The following are available online at http://www.mdpi.com/1996-1944/11/12/2540/s1, File S1: Geometrical and surface characterization report for FDM, DLP, and SLA under High hydrostatic high-pressure. (Figure S1: Example of the specimens manufactured with (a) DLP, (b) FDM and (c) SLA technologies, used for the surface and geometrical characterization, Figure S2: Alicona machine surface characterization sample images, Table S1: Measurements with digital caliper before HHP processing. Dimensions are in mm, Table S2: Measurements taken with the Alicona before HHP processing on DLP sample #1, Table S3: Measurements with digital caliper after HHP processing. Dimensions are in mm, Table S4: Measurements taken with the Alicona after HHP processing on DLP sample #1. Dimensions are in mm, Table S5: Caliper-measured dimensions changes after HHP processing. Absolute maximum values shown in bold, Table S6: Dimensions changes measured for DLP sample #1 on the Alicona machine. Dimensions are in mm.)
