# Supplementary material for: Hydrostatic High-Pressure Post-Processing of Specimens Fabricated by DLP, SLA, and FDM: An Alternative for the Sterilization of Polymer-Based Biomedical Devices
Source: Materials (Basel). 2018 Dec 13;11(12):2540. doi: 10.3390/ma11122540 (PMC6316578; doi:10.3390/ma11122540)
Supplement: Supplementary file 1 [file materials-11-02540-s001.pdf]

materials

Article

Hydrostatic High-Pressure Post-Processing of Specimens Fabricated by DLP, SLA, and FDM: An Alternative for the Sterilization of Polymer-Based Biomedical Devices

José A. Robles Linares-Alvelais [1,2,†], J. Obedt Figueroa-Cavazos [1], C. Chuck-Hernandez [3], Hector R. Siller [4], Ciro A. Rodríguez [1,2] and J. Israel Martínez-López [1,2,*,†]

1 Department of Mechanical Engineering and Advanced Materials, Tecnologico de Monterrey, NL, 64849, Mexico; a01226825@tec.mx (J.A.R.L.-A.); obedt.figueroa@tec.mx (J.O.F.-C.); cristina.chuck@tec.mx (C.C.-H.); ciro.rodriguez@tec.mx (C.A.R.)
2 Laboratorio Nacional de Manufactura Aditiva y Digital (MADiT), Apodaca,NL, 66629, México
3 Centro de Biotecnología FEMSA, Tecnologico de Monterrey,NL, 64849 , Mexico
4 Department of Engineering Technology, University of North Texas. 3940 N. Elm. St., Denton, TX 76207, USA; hector.siller@unt.edu
* Correspondence: israel.mtz@tec.mx; Tel.: +52-8183582000
† These authors contributed equally.

The 3D-printed probes used for the surface and geometrical characterization are shown in Figure 1. Figure 2 contains the Alicona machine obtained surface characterization images example and tables 1 through 6 show the dimensions before and after the HHP processing.

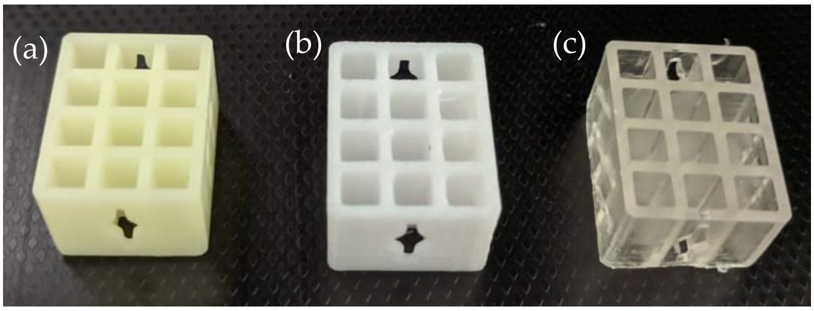

Figure S1. Example of the specimens manufactured with (a) DLP, (b) FDM and (c) SLA technologies, used for the surface and geometrical characterization.

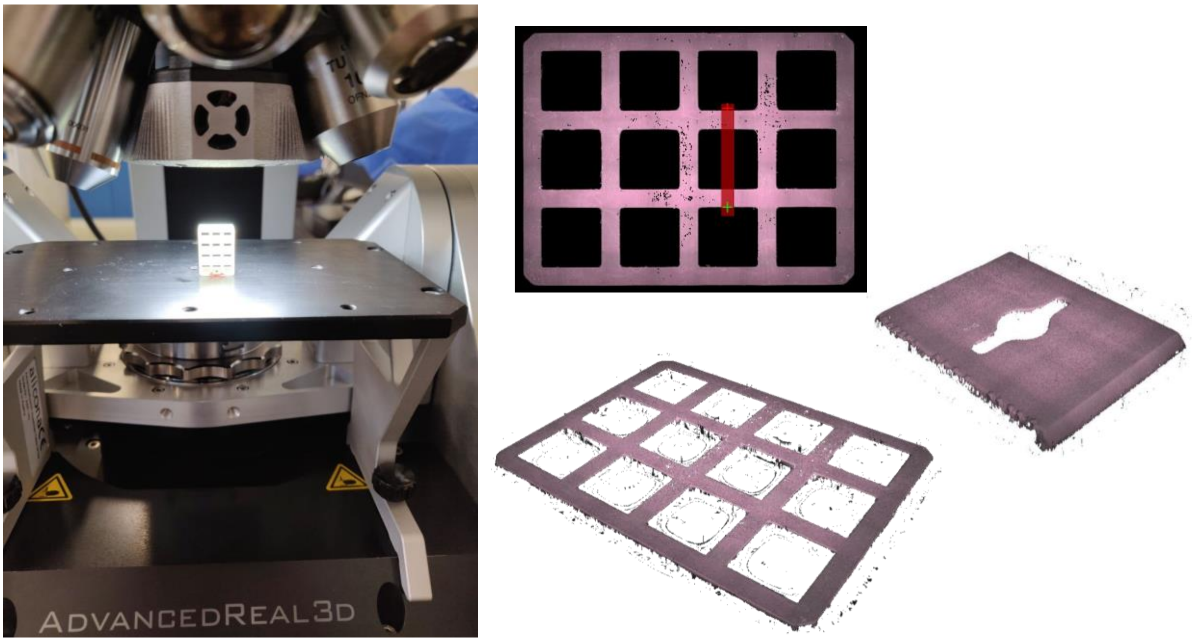

Figure S2. Alicona machine surface characterization sample images.

Table S1. Measurements with digital caliper **before** HHP processing. Dimensions are in mm.

Dimension	DLP			FDM			SLA		
	Sample 1	Sample 2	Sample 3	Sample 1	Sample 2	Sample 3	Sample 1	Sample 2	Sample 3
x	17.88	17.91	17.92	19.08	19.09	19.13	19.33	19	19.33
y	13.68	13.68	13.7	14.71	14.63	14.67	14.29	14.19	14.33
z	10.42	10.39	10.48	10.39	10.35	10.39	10.22	10.27	10.47
t_1	0.96	0.98	0.99	1.04	1	1.06	0.99	1.01	0.99
t_2	0.98	1.02	0.99	1.03	1.02	1.06	1.05	1.06	1.02
t_3	0.98	1	1.02	1.04	1.04	1.06	1.07	1.04	1.03
t_4	0.98	0.98	1.01	1.04	1.05	1.07	1.05	0.99	1.05
t_5	0.96	0.98	0.97	1.02	1.03	1.06	0.98	0.96	0.98
L_1	6.65	6.64	6.59	6.85	6.9	6.85	6.99	6.55	7
D_1	2.75	2.78	2.78	2.99	2.96	2.9	2.99	2.95	2.99
L_2	6.6	6.66	6.6	6.89	6.87	6.86	7.06	6.96	7.03
D_2	2.75	2.75	2.78	2.97	2.99	2.89	3.03	2.95	3.01

Table S2. Measurements taken with the Alicona **before** HHP processing on DLP sample #1. Dimensions are in mm.

Dimension	Measure
x	17.836
y	13.625
a_1	3.319
a_2	3.300
a_3	3.280
a_4	3.297
b_1	3.242
b_2	3.217
b_3	3.244
b_4	3.262

Table S3. Measurements with digital caliper **after** HHP processing. Dimensions are in mm.

Dimension	DLP			FDM			SLA		
	Sample 1	Sample 2	Sample 3	Sample 1	Sample 2	Sample 3	Sample 1	Sample 2	Sample 3
x	17.88	17.93	17.93	19.09	19.1	19.13	19.33	19.01	19.34
y	13.68	13.68	13.69	14.7	14.64	14.67	14.31	14.2	14.34
z	10.42	10.41	10.48	10.38	10.36	10.38	10.2	10.25	10.47
t_1	0.96	0.97	0.99	1.04	1.01	1.06	1	1.01	0.98
t_2	0.98	1.02	0.98	1.03	1.02	1.05	1.04	1.06	1.02
t_3	0.98	1	1.02	1.04	1.05	1.06	1.08	1.03	1.04
t_4	0.98	1	1	1.04	1.05	1.06	1.06	0.99	1.05
t_5	0.97	0.99	0.97	1.03	1.03	1.05	1	0.95	0.99
L_1	6.68	6.62	6.59	6.85	6.89	6.85	6.98	6.55	7
D_1	2.73	2.75	2.78	2.99	2.98	2.93	2.99	2.94	3.01
L_2	6.59	6.67	6.59	6.87	6.87	6.84	7.07	6.95	7.02
D_2	2.74	2.76	2.77	2.98	3	2.88	3.03	2.95	3.03

Table S4. Measurements taken with the Alicona **after** HHP processing on DLP sample #1. Dimensions are in mm.

Dimension	Measure
x	17.869
y	13.644
a_1	3.301
a_2	3.283
a_3	3.294
a_4	3.276
b_1	3.272
b_2	3.235
b_3	3.254
b_4	3.237

Table S5. Caliper-measured dimensions changes after HHP processing. Absolute maximum values shown in **bold.**

Dimension	DLP			FDM			SLA		
	Sample 1	Sample 2	Sample 3	Sample 1	Sample 2	Sample 3	Sample 1	Sample 2	Sample 3
x	0.00%	0.11%	0.06%	0.05%	0.05%	0.00%	0.00%	0.05%	0.05%
y	0.00%	0.00%	-0.07%	-0.07%	0.07%	0.00%	0.14%	0.07%	0.07%
z	0.00%	0.19%	0.00%	-0.10%	0.10%	-0.10%	-0.20%	-0.19%	0.00%
t_1	0.00%	-1.02%	0.00%	0.00%	1.00%	0.00%	1.01%	0.00%	-1.01%
t_2	0.00%	0.00%	-1.01%	0.00%	0.00%	-0.94%	-0.95%	0.00%	0.00%
t_3	0.00%	0.00%	0.00%	0.00%	0.96%	0.00%	0.93%	-0.96%	0.97%
t_4	0.00%	**2.04%**	-0.99%	0.00%	0.00%	-0.93%	0.95%	0.00%	0.00%
t_5	1.04%	1.02%	0.00%	0.98%	0.00%	-0.94%	**2.04%**	-1.04%	1.02%
L_1	0.45%	-0.30%	0.00%	0.00%	-0.14%	0.00%	-0.14%	0.00%	0.00%
D_1	-0.73%	-1.08%	0.00%	0.00%	0.68%	**1.03%**	0.00%	-0.34%	0.67%
L_2	-0.15%	0.15%	-0.15%	-0.29%	0.00%	-0.29%	0.14%	-0.14%	-0.14%
D_2	-0.36%	0.36%	-0.36%	0.34%	0.33%	-0.35%	0.00%	0.00%	0.66%

Table S6. Dimensions changes measured for DLP sample #1 on the Alicona machine. Dimensions are in mm.

Dimension	Measure
x	0.183%
y	0.142%
a_1	-0.54%
a_2	-0.52%
a_3	0.43%
a_4	-0.64%
b_1	0.93%
b_2	0.56%
b_3	0.31%
b_4	-0.77%
